# Beclin-1-mediated Autophagy Protects Against Cadmium-activated Apoptosis via the Fas/FasL Pathway in Primary Rat Proximal Tubular Cell Culture

**DOI:** 10.1038/s41598-017-00997-w

**Published:** 2017-04-20

**Authors:** Gang Liu, Yan Yuan, Mengfei Long, Tongwang Luo, Jianchun Bian, Xuezhong Liu, Jianhong Gu, Hui Zou, Ruilong Song, Yi Wang, Lin Wang, Zongping Liu

**Affiliations:** 1 College of Veterinary Medicine, Yangzhou University, 12 East Wenhui Road, Yangzhou, 225009 People’s Republic of China; 2 Jiangsu Co-innovation Center for Prevention and Control of Important Animal Infectious Diseases and Zoonoses, Yangzhou, 225009 People’s Republic of China; 3 Jiangsu Key Laboratory of Zoonosis, Yangzhou, China; 4 College of Animal Science and Veterinary Medicine, Shandong Agricultural University, Daizong Road No. 61, Tai’an, 271018 People’s Republic of China

## Abstract

The Fas/FasL signaling pathway is one of the primary apoptosis pathways, but the involvement and regulatory mechanism of this pathway by autophagy remain unclear. Here we demonstrated that cadmium (Cd) activated the Fas/FasL apoptosis pathway in rat proximal tubular (rPT) cells; this was accompanied by simultaneous activation of autophagy resulted in reduced apoptosis. In this model, we induced autophagy through RAPA and further demonstrated that autophagy protects against activation of Fas/FasL signaling and apoptosis. The antiapoptotic effect of autophagy was blocked by 3-MA, an autophagy inhibitor. The interactions between Beclin-1 and Fas, FasL, FADD, caspase-8 and BID/tBID were relatively weak, with the exception of cleaved caspase-8, indicated that minimal interactions between these proteins and Beclin-1 are involved in maintaining the balance of autophagy and apoptosis. Beclin-1 precipitated with cleaved caspase-8 in a dose-dependent mannter, and the expression was increased by siRNA against Beclin-1. These data suggested that Beclin-1-mediated autophagy impairs the expression and function of cleaved caspase-8 to protect against Cd-induced activation of apopotosis through Fas/FasL signaling pathway.

## Introduction

Cd is an occupational hazard and environmental pollutant which can cause a broad range of physiological, biochemical and behavioral dysfunctions^1^. Cd has an extremely long biological half-life. Unlike other complex organic pollutants, Cd cannot be degraded by microorganisms, enters the food chain through contact with Cd in paints, fertilizers, cosmetics, automobiles, and batteries resulting in Cd accumulation in ecosystems^2^. Cd exhibits multi-organ toxicity in the heart, brain, liver, bone, and kidney^3^. The kidney is the primary site for initial Cd accumulation; especially proximal tubule cells, which are very sensitive to Cd-induced damage^4^. The nephrotoxicity of Cd has been extensively studied and widely reported in literatures, and there is growing evidence that apoptosis and autophagy are the fundamental molecular mechanisms of Cd nephrotoxicity^5–7^.

The Fas/FasL (Fas ligand) pathway is a key regulator of apoptosis. The Fas (APO-1; CD95) antigen is a type I cell surface glycoprotein that transduces apoptotic signals after interaction with the Fas ligand. Fas belongs to the TNF receptor superfamily, and has a molecular mass that ranging from 43 to 52 kDa. Fas-induced apoptosis promotes parenchymal cell damage in liver disease, glomerular injury and acute renal failure^8–10^. FasL is an extensively glycosylated, 36 to 40 kDa type II membrane protein, and functions to induce apoptosis through cross-linking of the death-inducing receptor Fas. FasL expression is not restricted to activated T cells and natural killer cells, and is also expressed in the testis, small intestine, lung, and kidney^11, 12^. Membrane-bound Fas (mFas) has a single membrane-spanning domain, and Fas also exists as a soluble molecule (sFas) arising from alternatively spliced mRNA^13, 14^. sFas may prevent Fas-mediated apoptosis by blocking the interaction between mFas and FasL^13, 15^.

Autophagy is a controlled process by which cells degrade parts of their own cytoplasm, organelles, and other macromolecules in lysosomes to maintain homeostasis as an adaptative response to stress and adverse conditions^16, 17^. While it is known that the interaction between autophagy and apoptosis is at least partially regulated by Beclin-1 and Bcl-2, the precise role of autophagy during apoptosis is unclear^18^. Beclin-1 is a critical regulator of autophagy. Overexpression of Beclin-1 induces autophagy in mammalian cells^17^, and knockout of the Beclin-1 gene results in embryonic lethality in mice^19^.

It has been shown that Cd exposure increases Beclin-1 expression in rat cerebral cortical neurons^20^, as well as in the kidney of purse red common carp (*Cyprinus carpio*)^21^. Moreover, it has been reported that the Fas/FasL signaling pathway is activated by Cd in renal cells and other cell types^22, 23^. Several studies have focused on the role of Fas or FADD during autophagy^24–27^. Besirli *et al*.^24^ found that both autophagy and the Fas/FasL signaling pathway are activated during retinal detachment, indicating a role for Fas/FasL signaling in regulating photoreceptor apoptosis. Autophagy can be induced by FADD silencing in human breast cancer cells; this may be regulated by the expression of Ras homolog^26^. Here, we focus on the relationship between autophagy and Fas/FasL signaling apoptosis pathway during Cd exposure in rPT cells.

## Results

### Cd induces activation of the Fas/FasL apoptosis pathway

**Figure 1:**
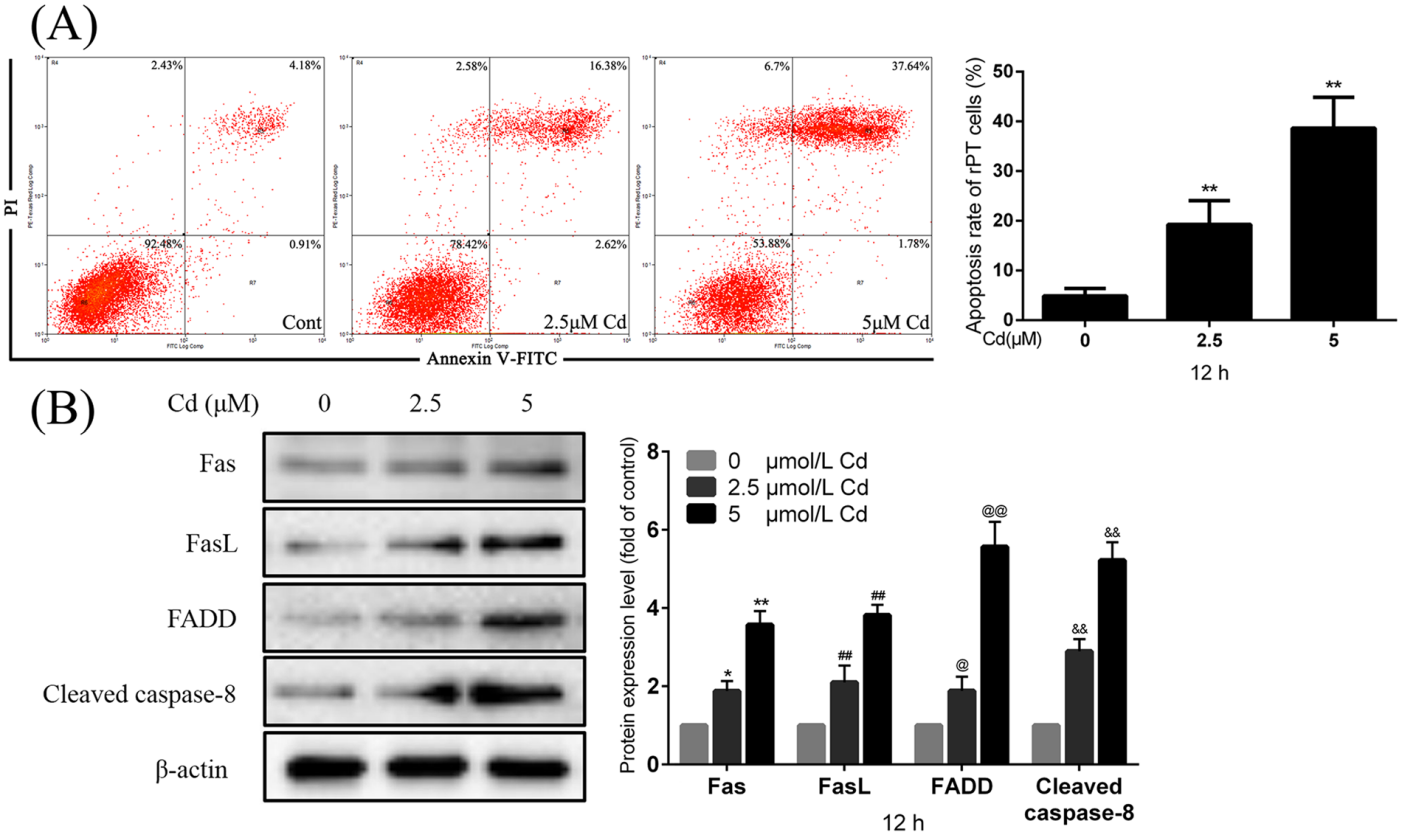
Effects of Cd on Fas/FasL signaling apoptosis pathway activation in rPT cells, measured by flow cytometry and western blot. **(A)** Cells were incubated with 0, 2.5 and 5 μM Cd for 12 h, and apoptosis markers were assessed using flow cytometry. Data are mean ± SEM of three separate experiments and each one performed in triplicate (n = 9). **(B)** Fas, FasL, FADD, and cleaved caspase-8 protein levels were assessed in rPT cells by western blot analysis after 12 h Cd treatment. The quantitative analysis was performed on the western blot results of four independent experiments (mean ± SEM, n = 4), respectively. ^*^ *P* < 0.05, ^**^ *P* < 0.01, ^##^ *P* < 0.01, ^@^ *P* < 0.05, ^@@^ *P* < 0.01 and ^&&^ *P* < 0.01.

Apoptosis of rPT cells was determined using flow cytometry (FCM). The number of apoptotic cells (early and late) was significantly enhanced in a dose-dependent manner after 12-h treatment with 2.5 μmol/L and 5 μmol/L Cd, 3.73- and 7.74-fold increased apoptosis over control cells, respectively (Fig. 1A). Proteins that are associated with the Fas/FasL apoptosis pathway were detected by immunoblotting, and Fas, FasL, FADD, and cleaved caspase-8 were significantly increased in a dose-dependent manner after 12-h treatment with 2.5 μmol/L and 5 μmol/L Cd (Fig. 1B). These results illustrated that the Fas/FasL apoptosis pathway can be activated by Cd in rPT cells.

### Cd reduce apoptosis in dose-dependent manner

**Figure 2:**
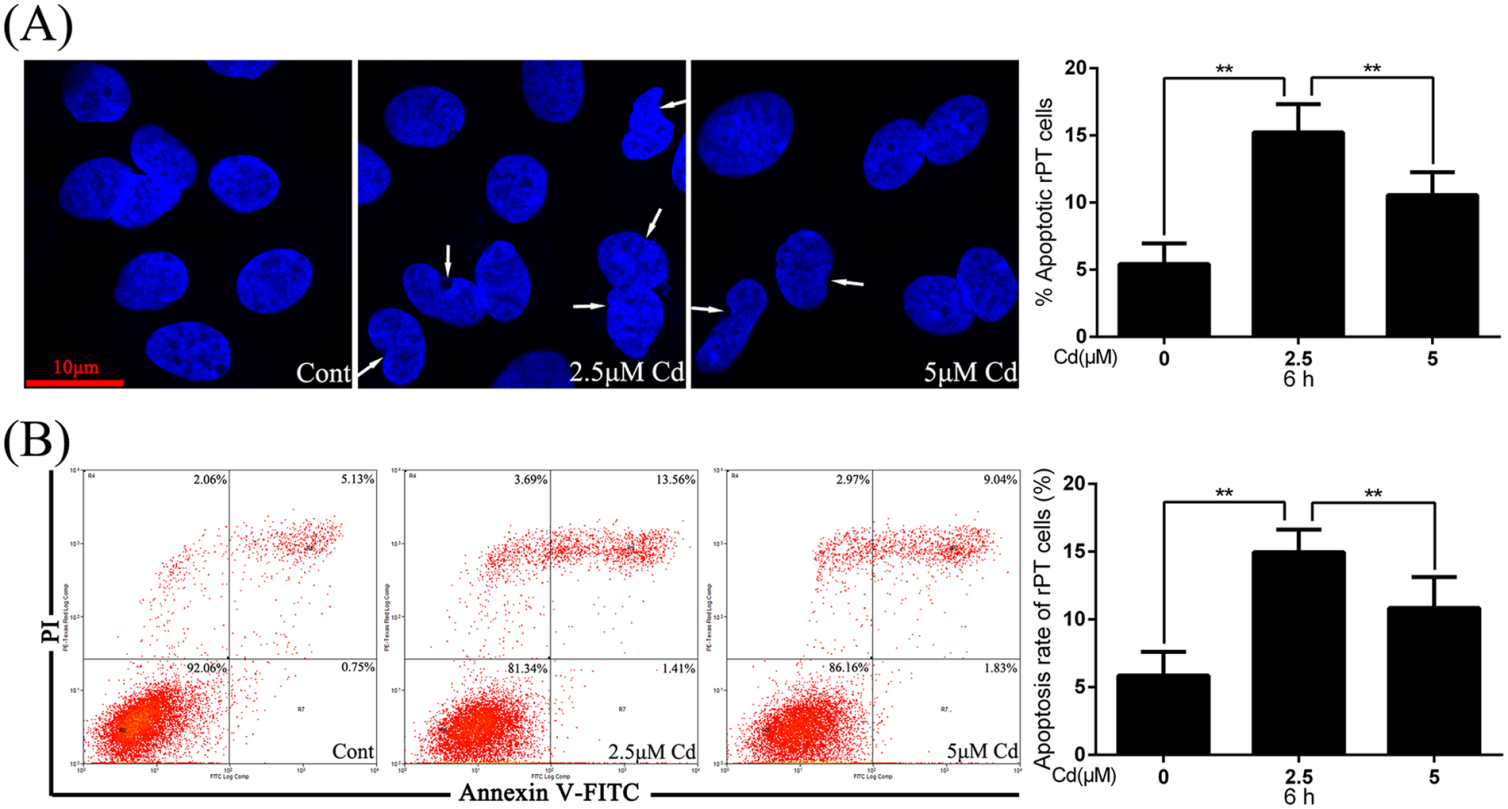
Effects of Cd on apoptosis of rPT cells. **(A)** Cells were incubated with 0, 2.5, and 5 μM Cd for 6 h and nuclear chromatin changes (apoptosis) were analyzed by confocal microscopy after DAPI staining. Changes of nuclei fragmentation with condensed chromatin are indicated by white arrows. The quantified results are expressed as mean ± SEM of three separate experiments, each performed in triplicate (n = 9). **(B)** rPT cells were treated with Cd for 6 h and apoptosus was assessed by flow cytometry. Data are presented as mean ± SEM of three separate experiments, each one performed in triplicate (n = 9). *P < 0.05 and **P < 0.01.

In control rPT cells, nuclear chromatin appeared regular with uniform staining throughout the entire nucleus. In contrast, rPT cells treated with Cd (2.5 μmol/L and 5 μmol/L) for 6 h exhibited morphological changes indicative of apoptosis: condensed chromatin appeared at the periphery of the nuclear membrane or appeared as a half-moon shape. Interestingly, the 5 μmol/L Cd treated group exhibited fewer changes in nuclei compared with the 2.5 μmol/L group (Fig. 2A). These observations were confirmed by FCM analysis, and 6-h treatment with 2.5 μmol/L and 5 μmol/L Cd resulted in a 2.55- and 1.85-fold increase in apoptosis compared to control cells, respectively. These results demonstrated that some protective mechanism may be activated after rPT cells are exposed to Cd for 6 h.

### Cd induces autophagy in rPT cells

**Figure 3:**
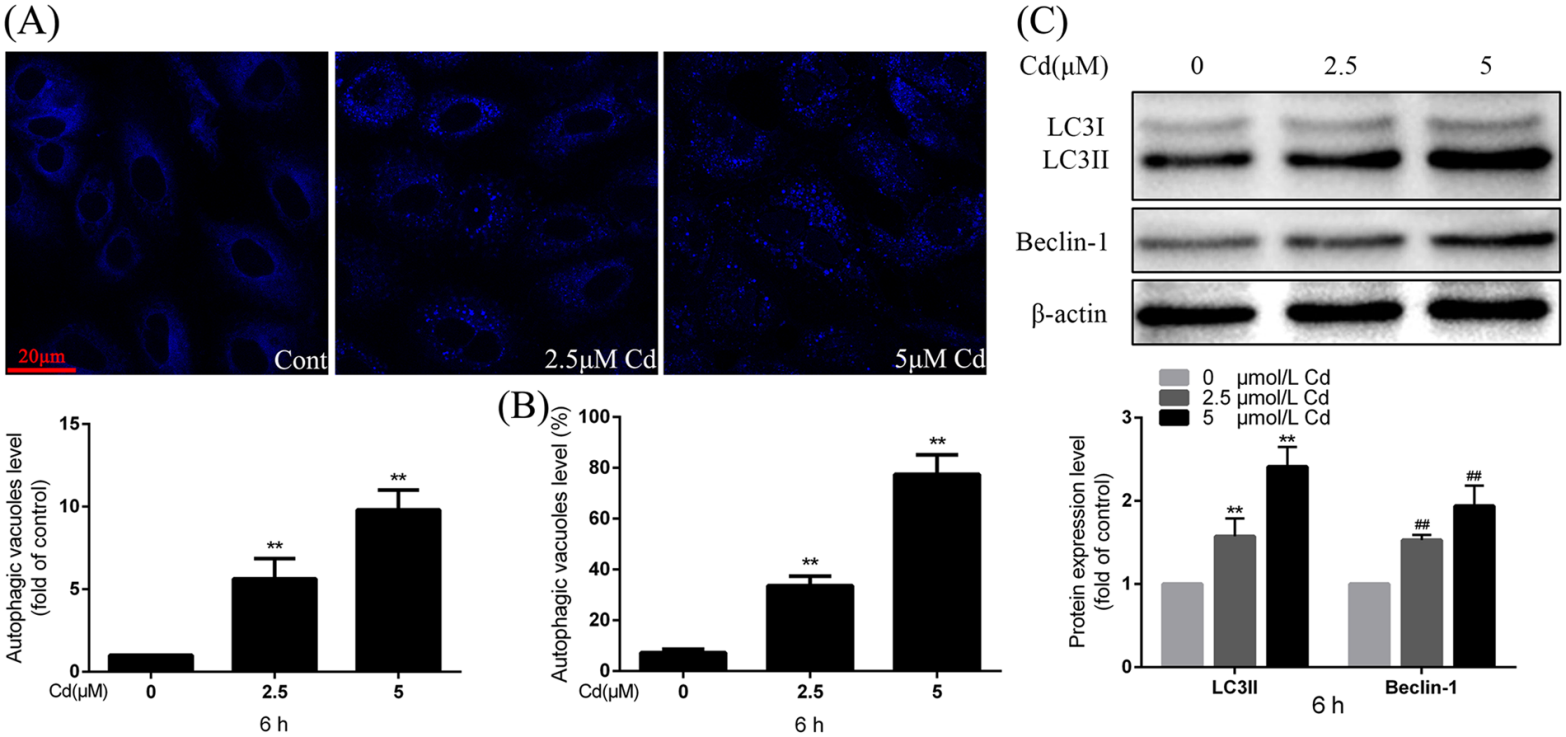
Effects of Cd treatment on activation of autophagy in rPT cells. **(A)** rPT cells were incubated with 0, 2.5, and 5 μM Cd for 6 h and autophagic vacuoles were analyzed by confocal microscopy after MDC staining. Fluorescence particles in the cytoplasm indicate autophagic vacuoles. The quantified analyses as assessed by MDC staining are expressed as mean ± SEM of three separate experiments; each experiment was performed in triplicate (n = 9). **(B)** rPT Cells were treated with Cd for 6 h and autophagy was assessed using flow cytometry. Data are mean ± SEM of three separate experiments, each experiment performed in triplicate (n = 9). **(C)** LC3II and Beclin-1 protein levels were assessed by western blot analysis after Cd treatment for 6 h. The quantitative analysis was performed on western blot images of four independent experiments (mean ± SEM, n = 4). ^**^ *P* < 0.01 and ^##^ *P* < 0.01.

rPT cells were treated with 2.5 μmol/L and 5 μmol/L Cd for 6 h and stained with dansylcadaverine (MDC); autophagy levels were assessed using LSCM and FCM. LSCM analysis demonstrated that Cd treatment enhances autophagy in rPT cells in a dose-dependent manner (Fig. 3A); this was confirmed by FCM, and it was determined that 2.5 μmol/L and 5 μmol/L Cd treatment induced a 4.59- and 10.56-fold increase in autophagy, respectively, compared to control cells (Fig. 3B). Immunoblotting indicated that expression of the autophagy marker and regulatory proteins, LC3II and Beclin-1, were significantly increased by Cd in a dose-dependent manner (Fig. 3C). These results demonstrate that Cd treatment simultaneously induces both apoptosis and autophagy.

### Autophagy blocks Cd-induced activation of the Fas/FasL apoptosis pathway

**Figure 4:**
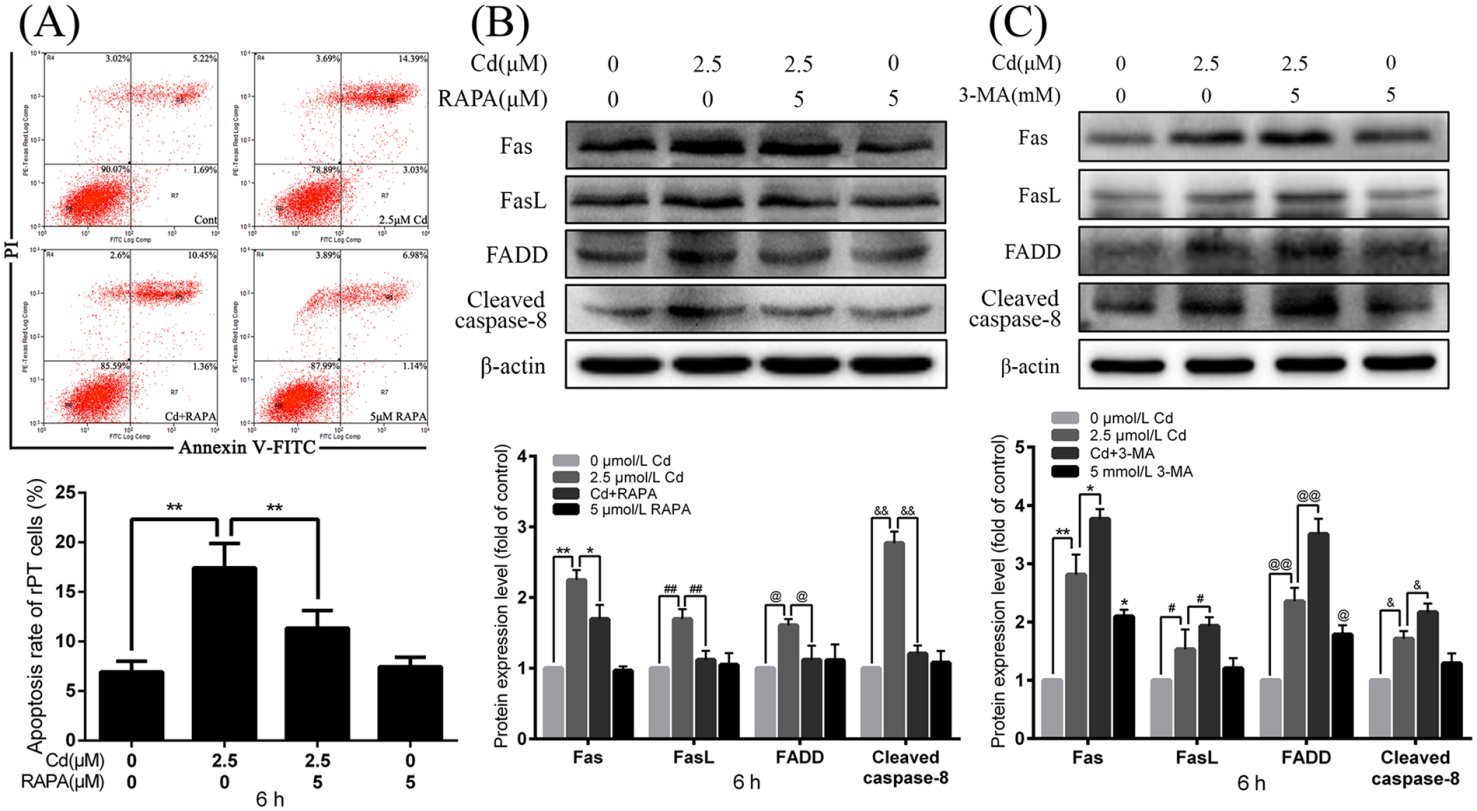
Effects of RAPA and 3-MA on Cd-induced apoptosis and activation of the Fas/FasL signaling pathway. **(A)** rPT cells were incubated with 2.5 μM Cd and/or 5 μM RAPA for 6 h, and apoptosis was assessed by flow cytometry. Data are presented as mean ± SEM of three separate experiments, each one performed in triplicate (n = 9). Fas, FasL, FADD and cleaved caspase-8 protein levels were assessed by western blot analysis after 6 h treatment with Cd and/or RAPA **(B)** and Cd and/or 3-MA **(C)**. The quantitative analysis was performed on western blot images of four independent experiments (mean ± SEM, n = 4). ^*^ *P* < 0.05, ^**^ *P* < 0.01, ^#^ *P* < 0.05, ^##^ *P* < 0.01, ^@^ *P* < 0.05, ^@@^ *P* < 0.01, ^&^ *P* < 0.05 and ^&&^ *P* < 0.01.

To investigate the effect of autophagy on Fas/FasL signaling pathway, an activator and inhibitor of autophagy, rapamycin (RAPA) and 3-Methyladenine (3-MA) were applied. FCM analysis demonstrated that treatment of rPT cells with 2.5 μmol/L Cd and 5 μmol/L RAPA induced a lower apoptosis rate compared with Cd treatment alone (Fig. 4A). Immunoblotting showed that Cd induced expression of Fas, FasL, FADD, and cleaved caspase-8 (Fig. 1B), while co-treatment with RAPA decreased expression of these proteins (Fig. 4B). Moreover, expression of Fas, FasL, FADD, and cleaved caspase-8 were increased significantly after rPT cells were treated with both 2.5 µmol/L Cd and 5 mmol/L 3-MA (Fig. 4C). These results illustrated that autophagy can block the Cd-activated Fas/FasL signaling apoptosis pathway.

### Beclin-1 interacts with members of the Fas/FasL signaling apoptosis pathway

**Figure 5:**
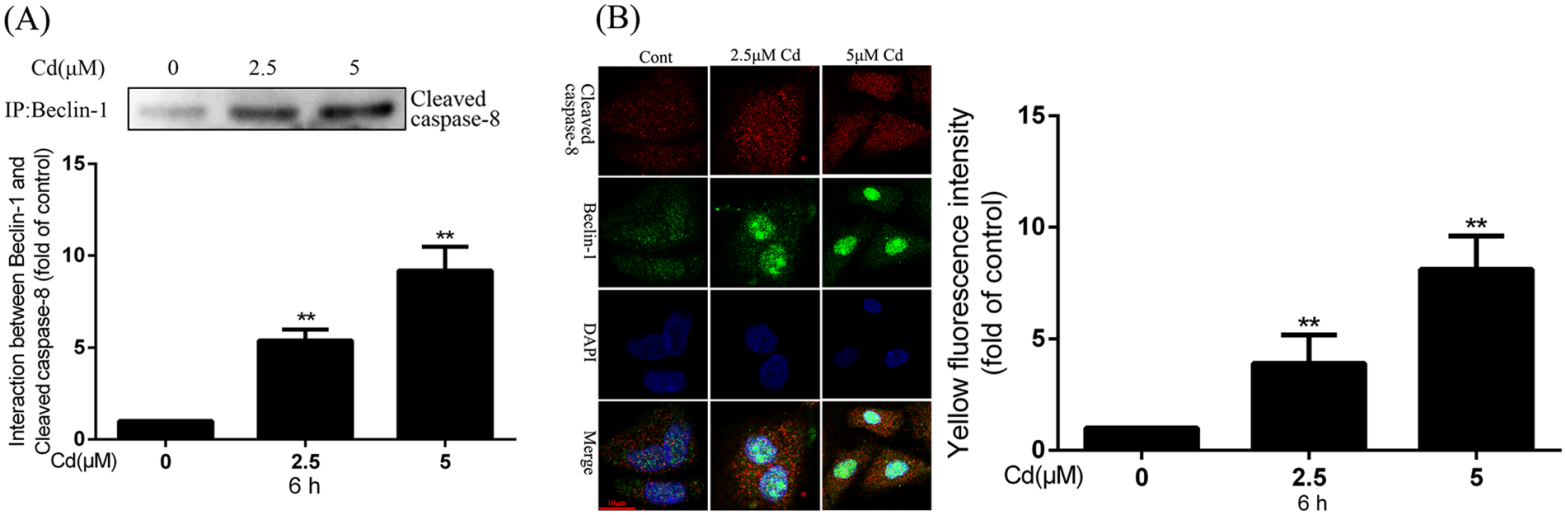
Interaction between Beclin-1 and cleaved caspase-8 after Cd treatment. **(A)** rPT cells were incubated with 0, 2.5, and 5 μM Cd for 6 h and the interaction between Beclin-1 and cleaved caspase-8 was analyzed by co-immunoprecipitation. The quantitative analysis was performed on western blot images of four independent experiments (mean ± SEM, n = 4). **(B)** Representative confocal images of co-localization of Beclin-1 and cleaved caspase-8; yellow fluorescence represent co-localization and the quantitative analysis performed with images of three independent experiments (mean ± SEM, n = 3). ^**^ *P* < 0.01.

To investigate the interaction between Beclin-1 and proteins in the Fas/FasL signaling apoptosis pathway, co-immunoprecipitation and immunofluorescence assays were applied. Co-immunoprecipitation showed that the interaction between Beclin-1 and cleaved caspase-8 was stronger folllowing 6-h Cd treatment (2.5 μmol/L and 5 μmol/L) in a dose-dependent manner (Fig. 5A). Immunofluorescence showed that co-localization of Beclin-1 and cleaved caspase-8 was also enhanced in a dose-dependent manner after 6-h Cd treatment (2.5 μmol/L and 5 μmol/L; Fig. 5B). No interaction or co-localization with Beclin-1 and other proteins in the Fas/FasL signaling apoptosis pathway was observed (data not shown). According to these results, it is possible that Beclin-1 plays a vital role in autophagy-dependent protection against Cd-induced apoptosis.

### Effect of Beclin-1 silence on cleaved caspase-8

**Figure 6:**
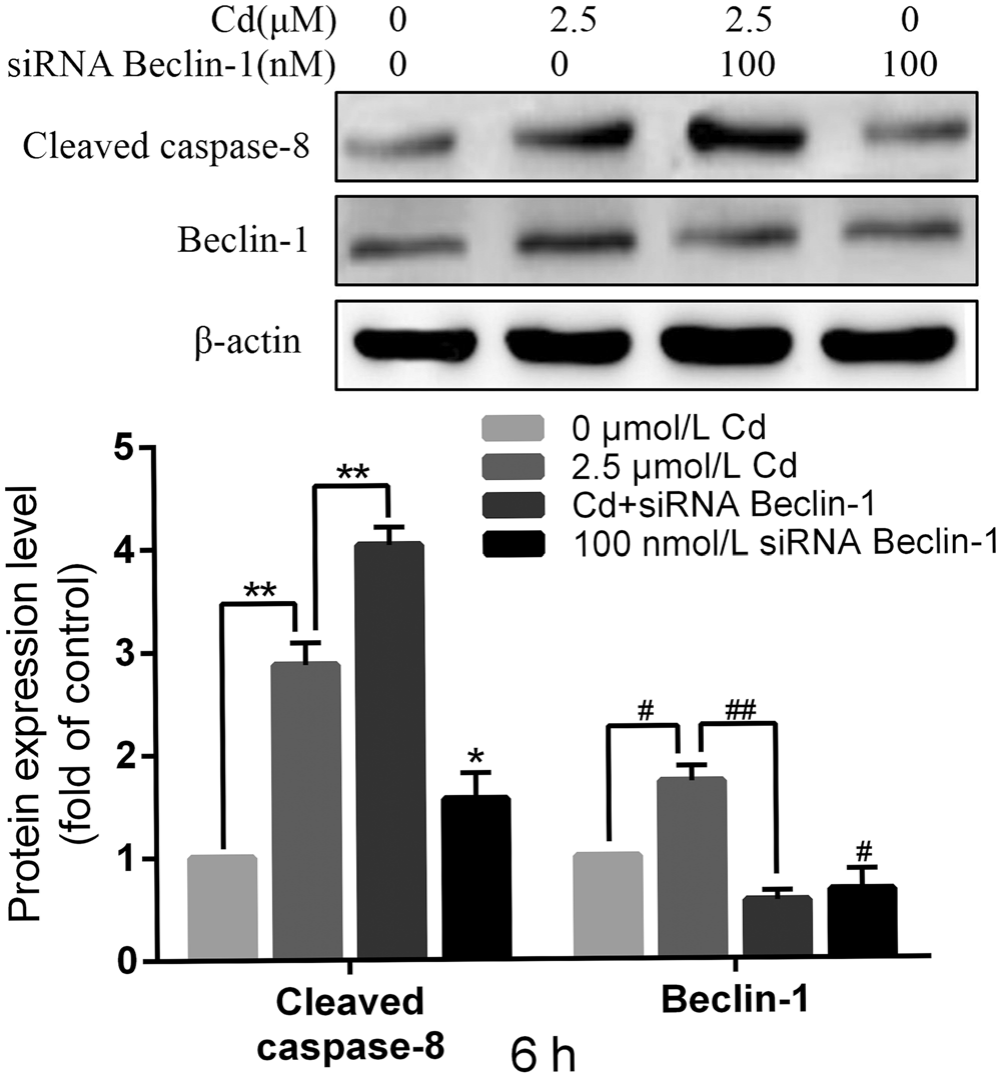
Effect of Beclin-1 knockdown on cleaved caspase-8 expression after Cd treatment. Cleaved caspase-8 and Beclin-1 protein levels were assessed by western blot analysis after 2.5 μM Cd and/or 100 nM siRNA Beclin-1 treatment 6 h. The quantitative analysis performed on western blot images from four independent experiments (mean ± SEM, n = 4). ^*^ *P* < 0.05, ^**^ *P* < 0.01, ^#^ *P* < 0.05 and ^##^ *P* < 0.01.

To further explore the relationship between Beclin-1 and cleaved caspase-8, siRNA targeting Beclin-1 and Z-IETD-FMK, a caspase-8 inhibitor, were applied. Immunoblotting showed that expression of cleaved caspase-8 was significantly increased 1.45-fold upon co-treatment with 100 nmol/L siRNA Beclin-1 and 2.5 μmol/L Cd (Fig. 6). However, compared to Cd treatment alone, co-treatment with Cd and Z-IETD-FMK resulted in no significant changes in Beclin-1 expression (data not shown). These results indicate that Beclin-1 and cleaved caspase-8 interact with each other and that cleaved caspase-8 was cleaved by Beclin-1.

## Discussion

rPT cells were applied as an *in vitro* model, we demonstrated that Cd induces activation of the Fas/FasL signaling apoptosis pathway. Expression of proteins in the Fas/FasL pathway were increased significantly after Cd exposure. Furthermore, activation of autophagy, indicated by autophagy vesicles and expression of autophagy related and regulatory proteins, was significantly increased after treatment with Cd. We further illuminated the role of Beclin-1 in Cd-induced activation of Fas/FasL signaling apoptosis pathway in rPT cells.

It has been previously suggested that autophagy and apoptosis can occur simultaneously, and autophagy can be induced by some inducers of apoptosis^28, 29^. The main event causing rPT cells death after treatment with Cd is apoptosis, which occurs in a dose-dependent manner in as little as 12 h^30^. In this study we confirmed that Cd activates the Fas/FasL signaling apoptosis pathway in rPT cells (Fig. 1). As it has been reported that the apoptosis rate decreases rapidly after treatment with Cd^31^, we assessed apoptosis rate in rPT cells after exposure to 2.5 μmol/L and 5 μmol/L Cd for 6 h, and we observed increased apoptosis in both the 2.5 μmol/L and 5 μmol/L Cd group, althouth the 5 μmol/L Cd treatment induced a lower apoptosis rate than the 2.5 μmol/L Cd did, but still elevated compared to the control (Fig. 2). This result was in concordance with a previous study by the Kondo group^31^, in which it was reported that CdCl_2_ exposure can induce the phosphorylation of cell survival-transcription factors, such as CREB, ATF-1, and c-Fos, to reduce apoptosis in HK-2 human renal proximal tubular cells.

It has been reported that arsenic trioxide induces not only apoptosis but also autophagy in leukemia cell lines through up-regulation of Beclin-1^32^. Therefore, we explored the possibility that autophagy may be activated as a protective mechanism in response to Cd treatment. We have previously shown that autophagy plays a protective role during Cd-induced apoptosis in BRL 3 A cells for a 6 h duration^33^ and in PC-12 cells for a 24 h duration^34^. According to our preliminary results, autophagy was activated from 2 to 18 h, and reached its peak after rPT cells were exposed to Cd for 6 h; this is in accord with our previous result in BRL 3A cells. We observed that co-treatment with Cd and RAPA resulted in a lower apoptosis rate (Fig. 3A) which increase the possibility of a protective role for autophagy, and led us to speculate that autophagy plays a protective role and to study its relationship with the Fas/FasL signaling apoptosis pathway activated by Cd in rPT cells.

Fas, FasL, FADD, and caspase-8 are four effector proteins upstream of the Fas/FasL signaling apoptosis pathway and act downstream to stimulate mitochondria mediated apoptosis by BID/tBID or to activate caspase-3 directly. The focus of most studies has been on the regulation of the relationship between autophagy and Fas or FADD^24–26, 35, 36^. Fluoride-induced apoptosis involved both extrinsic (Fas/FasL signaling) and intrinsic apoptosis pathways; it also simultaneously increases the number of autophagosomes and enhances the levels of autophagy marker LC3-II but not Beclin-1 in an *in vivo* experiment^37^. On the contrary, our results showed increases in both autophagosomes and expression levels of LC3-II and Beclin-1 after Cd treatment in rPT cells. The discrepency may be due to differences in toxicity and in the experimental model we employed. Another experiment on Retina–retinal pigment epithelium (RPE) separation found that Retina–RPE separation induced a Fas-dependent activation of autophagy, accompanied by increased ATG5 expression levels, intra-photoreceptor conversion of LC3-I to LC3-II, and inhibition of autophagy by 3-MA or siRNA ATG5 accelerated caspase-8 activation and photoreceptor TUNEL staining, demonstrating that autophagy plays a role in regulating the level of photoreceptor Fas associated apoptosis^24^. That result is very much in concordance with our present results. Moreover, we observed increased expression levels of Fas, FasL, FADD, and cleaved caspase-8 following co-treatment with 3-MA and Cd, compared with experimental controls. Treatment with the autophagy inducer, RAPA, showed that promoting autophagy decreased the expression of Fas/FasL pathway related proteins, which further proved that autophagy plays a constructive role in the apoptotic process. Beclin-1, the mammalian orthologue of yeast ATG6, has a central role in autophagic regulation, and has been linked to diverse biological processes including immunity, development, tumor suppression, and lifespan extension. In rats, lead (Pb) exposure induces neurotoxicity mediated by endoplasmic reticulum (ER) stress, which promotes expression of Beclin-1 and induces changes in the ratio of LC3II/LC3I which suggests that Pb can lead to autophagy^38^. Additionally, Cd triggers cell autophagy in skin epidermal cells, where the protein levels of Beclin-1 and LC3II formation are increased^39^. These studies confirm that Beclin-1 play a vital role in autophagic regulation.

Zhang *et al*.^25^ demonstrated that Fas mediates CH11-induced autophagy in HeLa cells, and suggest that autophagy is a protective mechanism against Fas-mediated apoptosis. They reported that caspase-8 has no influence on this process, whereas JNK activated by Fas participates in and promotes this autophagic process. In the present investigation, we demonstrated that Beclin-1, an autophagy regulatory protein, had no interaction with Fas; this may due to the fact that Fas belongs to a membrane protein which is not involved in the autophagy process and the same as FasL. How RAPA treatment decreased Fas and FasL expression level is unclear. One possible reason is some feedback signal existing; the mechanism underlying this observation needs to be further investigated. FADD has been described as a crucial element in many important biological processes including embryonic development, proliferation, cell cycle, innate immunity, tumor development, and autophagy^27, 40–42^. Many studies have focused on the role of FADD in autophagy^43, 44^. Thorburn *et al*.^27^ confirmed that autophagy is inactivated in FADD-DD-resistant epithelial cells and is involved in the caspase-independent cell death response to the FADD-DD signaling pathway in normal epithelial cells. And another study reported that autophagy was induced by FADD deficiency in MCF7 or MDA-231 cells but rescued by recovering Ras homolog enriched in brain (Rheb) expression^26^. Our study focuses on the interaction between FADD and Beclin-1. Co-immunoprecipitation revealed a very faint interaction between these two proteins (data not shown); this is the basic interaction required to maintain balance of autophagy and apoptosis. However, we detected a clear interaction between Beclin-1 and cleaved caspase-8, but not caspase-8, in a dose-dependent manner. Our results differ from the conclusion reached by Zhang *et al*., that caspase-8 has no influence on autophagic process^25^. However, our results are in line with the result that, during autophagy-mediated inhibition of apoptosis, Beclin-1 may be involved in the inhibition of Fas-mediated caspase-8 activation^24^. Various results obtained in different groups may be due to different toxic or different experimental models applied. We detected an interaction between Beclin-1 and BID/tBID, the result was similar to FADD. These data suggest that there is a very faint interaction effect that can help keep balance between autophagy and apoptosis.

In conclusion, our findings demonstrated that Cd activate both the Fas/FasL signaling apoptosis pathway and the autophagy pathway in rPT cells. Beclin-1 plays a crucial role in autophagy protect against Cd activated Fas-mediate rPT cells apoptosis. Our study suggests that Beclin-1 only interacts with cleaved caspase-8, not Fas, FasL and caspase-8, nor BID/tBID or mitochondrial mitochondria-related proteins, to execute its regulatory function.

## Materials and Methods

### Animals

All experimental procedures were conducted in accordance with the recommendations in the Guide for the Care and Use of Laboratory Animals of the National Research Council and were approved by the Animal Care and Use Committee of Yangzhou University (Approval ID: SYXK (Su) 2007 - 0005). Sprague-Dawley rats weighing between 180 g and 200 g were obtained from the Comparative Medicine Centre of Yangzhou University (Yangzhou, China). Animals were housed individually on a 12 h light/dark cycle with unlimited standard rat food and double distilled water (DDW). All surgeries operations were performed under sodium pentobarbital anesthesia, and all efforts were made to minimize any suffering experienced by the animals used in this study.

### Reagents

Dulbecco’s modified Eagle’s medium (DMEM)-F12 (1:1), Opti-MEM I Reduced Serum Medium, fetal bovine serum (FBS), trypsin-EDTA, collagenase IV and Lipofectamine 3000 Transfection Reagent were obtained from Thermo Fisher Scientific (Waltham, MA USA). Cadmium acetate, 4′, 6-diamidino-2-phenylindole (DAPI), 3-Methyladenine (3-MA), Rapamycin (RAPA) and dimethylsulfoxide (DMSO) were from Sigma-Aldrich (St. Louis, MO USA). Z-IETD-FMK (abcam, ab141382) was from Abcam Ltd (Cambridge, MA USA). Accutase cell detachment solution and the annexin V–fluorescein isothiocyanate/propidium iodide (FITC/PI) apoptosis detection kit were from Becton-Dickinson (San Diego, CA USA). SignalSilence® Beclin-1 siRNA II (CST, #6246) was from Cell Signaling Technology (Danvers, MA USA). Primary antibodies used were: anti–Fas antibody (abcam, ab82419), anti–FasL antibody (abcam, ab15285), anti–FADD antibody (abcam, ab14533), anti–cleaved caspase-8 antibody (abcam, ab25901) and anti–Beclin-1 (abcam, ab62557) were from Abcam Ltd (Cambridge, MA USA); anti–LC3 (Sigma, L7543) were from Sigma-Aldrich (St. Louis, MO USA) and anti–BID antibody (Novus, NB100-56106) was from Novus Biologicals (Littleton, CO USA); anti–caspase-8 antibody (Biorbit, orb88038) was from Biorbyt Ltd (San Francisco, CA USA). All secondary antibodies were from Beijing Zhongshan Golden Bridge Biotechnology (Beijing, China). And all other chemicals were purchased from Sigma-Aldrich, USA.

### Cell culture and Cd treatment

Briefly, intraperitoneal injection of sodium pentobarbital (2%, 0.31 ml/100 g) was used to anesthetize SD rats (from the Comparative Medicine Centre of Yangzhou University) with body weights between 180 g and 200 g. Cervical dislocation was performed to confirm death after the rats were completely anesthetized. Rats were transferring to sterile worktable after soaking in 75% alcohol for 2 minutes. The abdominal cavity was opened and kidneys were removed aseptic conditions. Isolation, identification and culture of rPT cells was performed as previously described^45^. Primary rPT cells and subcultures were cultured in DMEM/F12 supplemented with 15% FBS, 100 U/mL penicillin, 100 μg/mL streptomycin, and 0.25 g/L glutamine at 37 °C in 95% air and 5% CO_2_. rPT cell identity was confirmed by alkaline phosphatase antibody staining against specific proximal tubular antigens. The purity of the isolated primary rPT cells was >95%; the cells were subcultured using trypsin-EDTA digestion (0.25% and 0.02%). rPT cells cultured for 12 h had the highest viability (according to the growth curve, data not shown). A stock solution of Cd acetate was dissolved in sterile ultrapure water and 2.5 μmol/L and 5 μmol/L Cd were applied in present study according to the doses of Cd in a previous study^46^. The autophagy inhibitor 3-MA was dissolved in sterile ultrapure water and RAPA was dissolved in DMSO to make the stock solutions; stocks were filtered and stored at −20 °C, then diluted to working solution prior to use. The final concentration of DMSO was <0.01% and has no effect on autophagy and cell viability (data not shown).

### DAPI and MDC staining

Morphology of apoptotic cell nuclei was detected by staining with the DNA binding fluorochrome DAPI (4′,6-diamidino-2-phenylindole). rPT cells (2 × 10^5^ cells per well) were seeded on sterile cover slips placed in 24-well plates. After 12-h treatment with 0, 2.5, and 5.0 μmol/L Cd, cells were washed three times with ice cold PBS and fixed with 4% paraformaldehyde (PFA) for 10 min at room temperature. Following fixation, cells were incubated with DAPI staining solution (50 mM in PBS) for 10 min in the dark at room temperature. After three washes with PBS, the cells were viewed under a laser scanning confocal microscope (TCS SP8 STED; Wetzlar, Hessen, GER) at an excitation wavelength of 358 nm. Following induction of autophagy, rPT cells were incubated with 50 mmol/L MDC in PBS for 10 min at 37 °C. After incubation, the cells were washed three times with PBS and immediately visualized using a Lecia laser scanning confocal microscope (TCS SP8 STED; Wetzlar, Hessen, GER) at an excitation wavelength of 355 nm. To assess the extent of Cd-induced apoptosis and autophagy, 200 cells were randomly selected to record representative apoptosis and autophagy data for every experimental batch; each experimental group was assayed in triplicate.

### Immunofluorescence analysis

For immunofluorescence (IF) analysis, rPT cells were seeded on sterile cover slips placed in 24-well plates at a density of 2 × 10^5^ cells/well. After treatments, rPT cells were fixed with 4% PFA, permeabilized by exposure for 10 min to 0.5% TritonX-100 and placed in blocking solution (5% BSA) for 1 h. Cells were then exposed to primary antibodies overnight at 4 °C, followed by incubation with fluorecscent secondary antibodies at room temperature for 1 h in the dark. Cells were washed three times with ice cold PBS, were then fixed on slides with 50% glycerin, and were observed under a laser scanning confocal microscope (TCS SP8 STED; Wetzlar, Hessen, GER). Images for colocalization analysis were assessed using the JaCoP plugin in ImageJ after thresholding of individual frames. All colocalization calculations were performed on three independent experiments with 50 cells 1 per condition in each experiment^47^.

### Cell fraction preparation

After treatment, rPT cells were harvested using cell scrapers and were washed twice with ice cold PBS. To obtain the mitochondrial and cytosolic protein or membrane and cytosolic protein extracts, the harvested cells were subfractionized in homogenization buffer. The mitochondrial and cytosolic fractions were isolated with the method described by Jayanthi *et al*.^48^, and membrane and cytosolic fractions were isolated with CelLytic™ MEM Protein Extraction Kit according to the manufacturer’s protocol.

### Co-immunoprecipitation assay

rPT cells were harvested by Accutase™ Cell Detachment Solution. After cells were washed twice with ice cold PBS and sonicated, cell lysates were centrifuged at 12,000 rpm for 10 min at 4 °C. Supernatant was collected and the total protein concentration (>400 μg per sample) was adjusted to 1 μg/μl. Protein lysates were incubated with primary antibodies overnight at 4 °C in a roto-shaker. 50 μl of Protein G SureBeads (Bio Rad) was add into the complexes and incubated in a roto-shaker for 2 h at room temperature. The Protein G complexes were washed three times with PBST on a magnetic frame. After washes, samples were eluted with 20 μl glycine elution buffer (20 mmol/L, pH 2.0) and 2 μl phosphate buffer (1 mmol/L, pH7.4). The eluate was resuspended in lysis buffer and boiled for 5 min to prepare for western blot analysis.

### Western blot analysis

After cell fraction preparation and protein quantification with a bicinchoninic acid (BCA) protein assay kit (Beyotime, Shanghai, China), equal amounts of protein were separated by 8–15% sodium dodecyl sulfate–polyacrylamide gel electrophoresis (SDS-PAGE) and transferred to 0.22-μm or 0.45-μm polyvinylidene difluoride (PVDF) membranes. After transfer, membranes were blocked in 5% skim milk for 1 h at room temperature. The membranes were incubated overnight at 4 °C with the relevant primary antibodies. Membranes were then incubated with the appropriate secondary antibodies (1:5000) for 1 h at room temperature. Western blots were developed using enhanced chemiluminescence reagent. Protein levels were determined by computer-assisted densitometric analysis (GS-800 densitometer, Quantity One; Bio-Rad). The band volumes were determined by standard scanning densitometry with normalization of densitometry measures to β-actin. Each test was performed in triplicate.

### Flow cytometry analysis

rPT cells were seeded in 6-well plates and treated with Cd and/or inducer or inhibitor for 6 or 12 h when the cell fusion rate was 60–70%. Subsequently, the adherent cells were collected with Accutase™ Cell Detachment Solution by 5-min centrifugation at 1500 rpm. Each treatment group yielded 1.5 × 10^6^ cells, which were washed twice with ice cold PBS and incubated with fluorescent dyes for the flow cytometric analysis. The levels of apoptosis and autophagy were determined using a Beckman Coulter fluorescence-activated cell sorter (CyAn ADP 7; Brea, CA, USA).

### RNA interference

siRNA for Beclin-1 (Rat) was purchased from Cell Signaling Techology (CST, #6246). rPT cells were seeded in 6-well plates and transfected with 100 nmol/L Beclin-1 siRNA in 1 mL of medium containing Lipofectamine 3000 (Invitrogen) according to the manufacturer’s protocol, for 60 h prior to cell lysis.

### Statistical Analysis

Data from the present study are presented as mean ± SEM from at least three independent experiments with different batches of cells, and each experiment was performed in duplicate or triplicate, as indicated. Statistical comparisons were made using one-way analysis of variance (ANOVA; Scheffe’s F test) after ascertaining the homogeneity of variance between the treatments. All statistical data were analyzed using SPSS 19.0 (SPSS, Chicago, IL, USA). The critical value for statistical significance was *P* < 0.05.
